# The “Neglected Viruses” of *Taihu*: Abundant Transcripts for Viruses Infecting Eukaryotes and Their Potential Role in Phytoplankton Succession

**DOI:** 10.3389/fmicb.2020.00338

**Published:** 2020-03-06

**Authors:** Helena L. Pound, Eric R. Gann, Xiangming Tang, Lauren E. Krausfeldt, Matthew Huff, Margaret E. Staton, David Talmy, Steven W. Wilhelm

**Affiliations:** ^1^Department of Microbiology, The University of Tennessee, Knoxville, Knoxville, TN, United States; ^2^State Key Laboratory of Lake Science and Environment, Nanjing Institute of Geography and Limnology, Chinese Academy of Sciences, Nanjing, China; ^3^Department of Entomology and Plant Pathology, The University of Tennessee, Knoxville, Knoxville, TN, United States

**Keywords:** ssRNA, *Marnaviridae*, metatranscriptomic, harmful algal bloom, top-down, kill-the-winner

## Abstract

Drivers of algal bloom dynamics remain poorly understood, but viruses have been implicated as important players. Research addressing bloom dynamics has generally been restricted to the virus-infection of the numerically dominant (i.e. bloom forming) taxa. Yet this approach neglects a broad diversity of viral groups, limiting our knowledge of viral interactions and constraints within these systems. We examined hallmark virus marker genes in metatranscriptomic libraries from a seasonal and spatial survey of a *Microcystis aeruginosa* bloom in Lake Tai (*Taihu*) China to identify active infections by nucleocytoplasmic large DNA viruses (NCLDVs), RNA viruses, ssDNA viruses, bacteriophage, and virophage. Phylogenetic analyses revealed a diverse virus population with seasonal and spatial variability. We observed disproportionately high expression of markers associated with NCLDVs and ssRNA viruses (consistent with viruses that infect photosynthetic protists) relative to bacteriophage infecting heterotrophic bacteria or cyanobacteria during the height of the *Microcystis* bloom event. Under a modified kill-the-winner scheme, we hypothesize viruses infecting protists help suppress the photosynthetic eukaryotic community and allow for the proliferation of cyanobacteria such as *Microcystis*. Our observations provide a foundation for a little considered factor promoting algal blooms.

## Introduction

Harmful algal blooms (HABs) are extreme biological events that have detrimental environmental and socioeconomic effects on both fresh and salt waters. In particular, freshwater HAB events during the last two decades have posed threats to potable water supplies and socioeconomic resources for population centers (Qin et al., 2010; Steffen et al., 2014; Bullerjahn et al., 2016). Freshwater HABs commonly manifest as a summer bloom of cyanobacteria that follows from a winter/spring population of eukaryotic phytoplankton (Ke et al., 2008; Niu et al., 2011; Edgar et al., 2016). HAB research has historically focused on abiotic “bottom-up” controls, such as nutrient or temperature triggers that stimulate cyanobacterial growth (Tang et al., 2018). However, biological success can also arise during suppression of competitors, and there is less known about the decline of the eukaryotic population and factors that constrain these populations as cyanobacterial blooms form. And while grazing is often cited as the major driver of “top-down” regulation on communities (Vanderploeg et al., 2001), viruses also act as predators of algae.

Research over the past two decades has made it clear that viruses are central to ecosystem function (Wigington et al., 2016). There are many cases of direct viral modulation of phytoplankton populations in the literature, but these focus almost entirely on the lysis of dominant bloom-forming species (Hewson et al., 2001; Wilson et al., 2002; Tomaru et al., 2004; Brussaard et al., 2005; Steffen et al., 2017). In particular, there is less focus on numerically less-abundant protists that may be subject to virus-mediated suppression. In part this may be due to the general consensus that viruses of prokaryotes are the most abundant and active in aquatic systems (Weinbauer, 2004), or that many of the viruses infecting protists contain RNA genomes (Moniruzzaman et al., 2017) and are thus not detected by DNA-based metagenomics. These “neglected” viruses have the potential to control community dynamics, reducing the success of competitors. The “kill-the-winner” model invokes this important role of viral suppression of competitive hosts in microbial community assembly (Winter et al., 2010): however, the kill-the-winner model assumes equilibrium dynamics, and is historically thought of in the context of heterotrophic bacteria (Thingstad, 2000). Nevertheless, suppression of competing hosts by viruses, consistent with kill-the-winner, could be important in the selection of dominant, bloom-forming phytoplankton.

To explore the role of the “neglected” viruses, we examined metatranscriptomic data from a Lake Tai (China, or *Taihu* in Mandarin) bloom event during 2014. Lake Tai experiences yearly successions from photosynthetic protists to cyanobacteria (dominated by *Microcystis*) and therefore presents an opportunity to study the impact of viruses during and after that transition (Supplementary Figure 1; Ke et al., 2008; Niu et al., 2011). We assessed gene markers (Moniruzzaman et al., 2017; Stough et al., 2018) for active infections by nucleocytoplasmic large DNA viruses (NCLDVs), ss/ds RNA viruses, ssDNA viruses, as well as bacteriophage that were infecting non-*Microcystis* populations. In parallel, we characterized the putative host community structure. We detected a broad spatial and temporal richness of viruses, including a shift between the two dominant virus types (*Microcystis*-infecting phage and ssRNA viruses). In addition to observations of active eukaryotic viruses, determinations of putative host diversity and statistical co-occurrence analyses of “*who infects whom*” reveal potential advantages for *Microcystis* that account for part of its ability to form large bloom events in fresh waters.

## Materials and Methods

### Sample Collection

Samples were collected monthly (June to October) from a *Microcystis* spp.-dominated bloom in Lake Tai, China in 2014 at several stations across the northern parts of the lake as described previously (Krausfeldt et al., 2017; Stough et al., 2017). Biomass was collected by filtering between 25 and 180 mL (volume dependent on sample biomass density) of lake water through 0.2-μm nominal pore-size Sterivex^TM^ filters (EMD Millipore Corporation, Burlington, MA, United States) and immediately treated with ∼2 mL of RNA*later* (Qiagen) for preservation. Samples were collected in a manner consistent with the separation of bacteria and phytoplankton from dissolved materials: free virus particles generally pass through these filters. Environmental parameters and nutrient data were collected with each sample and have been reported elsewhere (Tang et al., 2018). Total RNA was extracted, quality checked, ribosomally reduced, and sequenced on the Illumina^TM^ HiSeq platform at HudsonAlpha Institute Genomic Services Laboratory (Huntsville, AL, United States) as previously described (Krausfeldt et al., 2017; Stough et al., 2017; Tang et al., 2018). Raw sequence data was retrieved from the HudsonAlpha servers and primarily handled in the CLC Genomics Workbench v. 10.1.1 suite (QIAGEN, Hilden, Germany). Quality scoring was used to remove reads with a score <0.3. Trimmed reads from all 35 samples were combined into a single file and assembled using MEGAHIT to reduce redundancy that can be seen within different contig fragments from identical genes (Li et al., 2015).

### Virus Detection

We employed a marker gene-based approach to discover viruses within the *Taihu* samples (Moniruzzaman et al., 2017; Stough et al., 2018). This protocol is publicly available (Pound and Wilhelm, 2019). A reference protein database was created from hallmark viral genes from a diverse range of taxonomic groups with sequences downloaded from the NCBI refseq database and Uniprot. Hallmark genes included the NCLDV major capsid protein (25 sequences), RNA virus RNA-dependent RNA polymerase (79 sequences), ssDNA viral replicase (11 sequences), and bacteriophage ribonucleotide reductase (1347 sequences) from characterized viral isolates. For virophage major capsid proteins, 9 sequences from virophage isolates as well as 7 from well-studied metagenomic assemblies were used. All data used to establish base phylogenies are available (Table 1 and Supplementary Material), and were selected to capture the known diversity in each sample (or as much known diversity as available in databases). Several marker genes were used to discover bacteriophage, including gp20, gp23, integrase, and ribonucleotide reductase, with the results of the latter presented in this paper. The other marker genes revealed no additional phage, nor did the phage finding tool, VirSorter (Roux et al., 2015). DNA-directed RNA polymerase beta subunit (Rpb1/RpoB) sequences (3,112 total) from microbial isolates were used as a gene marker to identify potential eukaryotic and prokaryotic hosts (Table 1 and Supplementary Table 1).

**TABLE 1:** Gene markers used in BLASTx for each virus type with reference.

**Target viral type**	**Marker gene**	**Reference used**
NCLDVs	Major capsid protein	NCBI refseq manually curated (modified from Moniruzzaman et al., 2017)
RNA viruses	RNA-dependent RNA polymerase	PF00680 v. 31
ssDNA viruses	Replicase/helicase	PF00910 v. 31
Virophage	Major capsid protein	NCBI refseq manually curated (Moniruzzaman et al., 2017)
Bacteriophage	Gp20, gp23, integrase, ribonucleotide reductase	PF06810, PF07068, PF00589, PF00317
Hosts	DNA-directed RNA polymerase	k03043 and k03006 (Kegg orthology)

*All sequences from pfam domains were viral.*

The assembled library was queried with each virus-specific reference protein database in command line BLASTx v.2.6.0+ with an e-value maximum of e^–10^ for viral candidates and e^–30^ for host candidates. A lower stringency was applied to potential viral candidates given the limited number of viral isolates in reference databases. Our separate stringency cutoffs for virus and hosts attempt to eliminate the implicit bias toward broader diversity in the hosts caused by the larger available database. A hidden Markov model search was used to validate our blast method but yielded 11 fewer viral candidates and provided no new candidates (data not shown). A custom python script was used to identify the coordinates of the aligned sequence within the query contig and extract only this region: these extracted components of the contigs were subsequently considered “viral candidates” (Gann et al., 2019). Only viral candidates greater than 150 nucleic acids were kept for further analyses. These remaining candidates were then queried against the NCBI refseq database, and any candidates with non-viral hits were removed from the analysis and considered false-positives. In preliminary work, we found that candidates less than 150 nucleic acids did not consistently produce positive blast hits to viral sequences in the refseq blast. Candidates were considered to represent “near complete” genes if they were larger than the smallest reference protein in length. Shorter candidates were considered and are discussed as “gene fragments.”

RNA virus candidates were further analyzed using the pfam v. 29 database to identify ORFs in the full-length contigs (contigs before extraction of target gene sequence). Contigs containing both structural and non-structural components were considered “near complete genomes” (data not shown) (Moniruzzaman et al., 2017).

### Viral Phylogeny and Expression

The hallmark gene reference proteins and “near complete” gene candidates were used to reconstruct maximum likelihood phylogenies as described in Moniruzzaman et al., 2017 (Supplementary Table 2). Gene fragment candidates were placed on trees using pplacer and subsequently visualized in iTOL v.4 (Matsen et al., 2010; Letunic and Bork, 2019). All reference proteins used to reconstruct phylogenies came from viruses that have been isolated in labs and sequenced, with the exception of some virophage sequences. *Mavirus*, *Sputnik*, and *Zamilon* remain the only virophage that have been isolated, purified and sequenced to date (La Scola et al., 2008; Fischer and Suttle, 2011; Gaia et al., 2014), while three *Chrysochromulina parva* Virus Virophages (CpVVs denoted Curly, Larry, and Moe) have been isolated but not purified beyond unialgal lysates (Stough et al., 2019). Several intact virophage have been assembled from metagenomic analyses and were employed here to make our phylogenetics more robust (Zhou et al., 2013, 2015).

Relative virus and host activity in each sample was quantified by mapping trimmed reads from each sample library to the extracted viral or host candidates using a 90% similarity fraction over a 90% length fraction in CLC Genomics Workbench 10.0 (Supplementary Table 3). The abundances for each viral type therefore only represent the expression of the singular target gene (and not other components of the contig). Transcript abundance data were normalized by the length of the extracted viral portion of the candidate contig and by the library size of that sample. Viral transcript expression was visualized using Heatmapper (Babicki et al., 2016). A non-metric multidimensional scaling (nMDS) plot using Bray–Curtis dissimilarity was generated in Primer7 and used to describe the influence of environmental factors on the abundance data (Clarke and Gorley, 2015).

### Cluster Analyses

We worked from the assumption that virus transcripts in cells represent active infections and that viruses require a host population to be present to generate these transcripts. Pearson correlation coefficients were established from square root transformed normalized transcript abundances using Primer 7 (Clarke and Gorley, 2015). A SIMPROF test (α = 0.5) was used to identify significant clusters of co-occurrences. Significant clusters containing both a putative host and putative virus candidate with correlation coefficients greater than 0.8 were visualized in Cytoscape (Smoot et al., 2011) with the nodes representing the viral or host candidate and the edges representing the strength of the correlation.

### Data and Code Availability

Raw sequencing data is publicly available in the MG-RAST database (Keegan et al., 2016) under the name “Lake_ *Taihu*_metatranscriptome_project.” Sequencing information and quality control details have been previously reported (Tang et al., 2018). Assembled data is also available in the MG-RAST database under the name “*Taihu* 2014 MegaHit Assembly 1.” The python script used to extract the aligned portion of blast hits is available in GitHub at https://github.com/Wilhelmlab/general-scripts/blob/master/Pound2019_Extract_aligned.py.

## Results and Discussion

Our analyses revealed an active and diverse virus community in the *Microcystis*-dominated HAB that occurred in *Taihu* during the 2014 summer sampling season. In total 8.42 × 10^8^ transcripts across 35 samples collected during the bloom season passed QA/QC assessments. Across all stations ∼70% of the transcripts were designated to be cyanobacterial in origin (Tang et al., 2018), highlighting the “bloom” nature of this data set. Of the residual transcripts, ∼1.92 × 10^5^ were associated with viruses. Phage assigned to groups known to infect *Microcystis* hosts comprised 47.9% of the total viral reads. The remaining 52.1% consisted of transcripts associated with a broad diversity of viruses. We assigned 827 unique viral candidate contigs to groups that included the NCLDVs, single-stranded and double-stranded RNA viruses, ssDNA viruses, non-*Microcystis* phage, and virophage.

**FIGURE 1:**
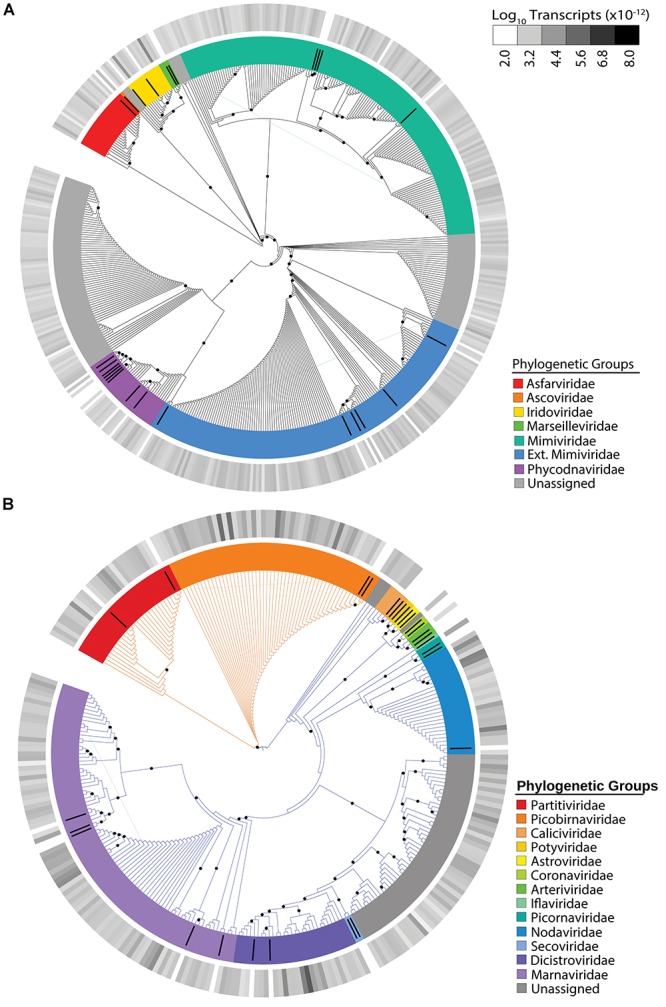
Maximum-likelihood phylogenetic placement of NCLDV candidates **(A)** and RNA virus candidates **(B)**. Reference proteins are denoted by black bars with colors indicating viral family (Supplementary Table 2). Orange branches indicate dsRNA viruses and blue branches indicate ssRNA viruses **(B)**. The outer heatmap ring indicates normalized transcript abundance for each candidate summed for all samples. Black dots represent bootstrap values >0.5.

The NCLDV group displayed the most richness (i.e. number of unique viral contigs) amongst the identified viral groups, with 457 candidates assigned to families (Figure 1A). The majority of these candidates were assigned to the “Extended-*Mimiviridae*” or the *Mimiviridae* clades, which have been associated to date with many marine algae (Claverie and Abergel, 2018). The most abundant NCLDV candidate contigs were assigned to the *Iridoviridae*, a NCLDV family whose members infect insects and cold-blooded vertebrates (Chinchar et al., 2009). Overall, NCLDVs made up 8.35% of the total reads in the virus dataset.

We also observed virophage signatures in our dataset, although with less richness (30 unique candidates) than the NCLDVs (Supplementary Figure 2). Most candidates were more closely related to an environmentally derived sequence identified as the Dishui Lake Virophage (DSLV) (Gong et al., 2016). Transcripts associated with virophage were the least abundant in our dataset, making up only 0.25% of the total viral reads or <0.0001% of the total reads.

The use of a metatranscriptomic data from size-selected (>0.2 μm) populations allowed us to detect infections by both DNA and RNA viruses, the latter of which should primarily be cell-associated. We detected 268 unique RNA virus candidates (Figure 1B). Overall, RNA viruses comprised 42.45% of the total virus transcripts, rivaling the 47.9% made up by *Microcystis* phage. Of the RNA virus candidates, 72% of the expression was assigned to ssRNA viruses and 28% to dsRNA viruses. The distribution between the number of single-stranded and double-stranded candidates was coincidentally identical, with 193 ssRNA virus candidates representing 72% of the richness and 75 dsRNA virus candidates representing 28% of the richness (Figure 1B). The dsRNA virus contigs belonged to *Partitiviridae* and *Picobirnaviridae*. Most of our ssRNA virus candidates belonged to the *Picornavirales* order, with the majority belonging to the *Marnaviridae* or *Dicistroviridae* families. *Marnaviridae* viruses, whose members include the *Chaetoceros tenuissimus* virus and the *Rhizosolenia setigera* virus, commonly infect marine diatoms (Nagasaki et al., 2004; Shirai et al., 2008). In our dataset, *Marnaviridae*-like viruses represent 12% of the total RNA virus reads, while *Dicistroviridae-*like viruses, which are typically associated with insect hosts (Valles et al., 2017), comprise 43% of our total RNA virus reads. Interestingly, the majority (86%) of the *Dicistroviridae*-associated transcripts come from a single candidate, which was highly expressed in most of our samples.

We took the opportunity to examine the RNA virus contigs to determine if any represented near or full-length virus genomes. Of our original 268 contigs, 16 were determined to contain a “near full-length” genome based on our parameters (Supplementary Figure 3). All of these viruses were single-stranded, and most belonged to *Marnaviridae* or *Nodaviridae*. Each meta-assembled virus contained both an RNA-dependent RNA polymerase and a structural gene, most commonly a capsid protein specific to the phylogenetic group.

As part of our classification efforts, we also observed evidence for both ssDNA viruses and dsDNA bacteriophage not associated with *Microcystis* spp. (“other” phage). However, neither group contributed much to the overall transcript abundance. The ssDNA viruses had only 17 unique candidates, which comprised only 0.42% of the total viral reads or <0.0001% of the total reads (Supplementary Figure 4). Eight candidates belonged to *Nanoviridae*, which typically infect plants. Ribonucleotide reductase markers indicated the presence of 14 unique candidates that were closely related to *Microcystis* phage. The remaining, “other,” phage had only 41 unique candidates, representing 0.63% of the total viral reads and 0.001% of the total reads (Supplementary Figure 5). The majority of the “other” phage belonged to *Caudovirales*, with several candidates most closely related to other cyanophages, such as *Synechococcus* phage.

**FIGURE 2:**
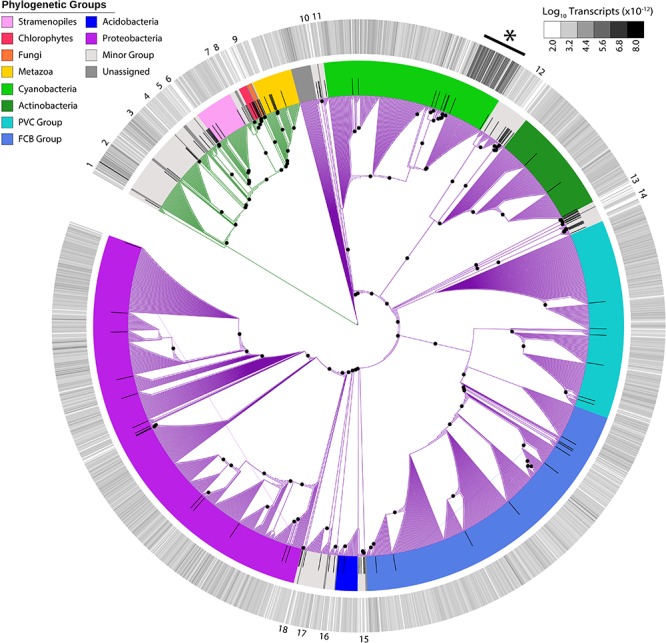
Maximum-likelihood phylogenetic placement of host candidates. Reference proteins are denoted by black bars. Green branches indicate eukaryotic hosts and purple branches indicate bacterial hosts. Colored ring indicates viral family and the outer heatmap ring indicates normalized transcript abundance for each candidate summed for all samples. Numbers on outer edge represent collapsed reference groups (Supplementary Table 2). Black dots represent bootstrap values >0.5. ^∗^*Microcystis*-like candidates.

In addition to the virus community, we examined eukaryotic and bacterial members of the putative host community. With 1,946 unique candidates, we observed a vast richness that spanned cellular phylogeny (Figure 2). Host transcripts associated with DNA-dependent RNA polymerase beta-subunit made up 0.06% of the total reads in the dataset. Candidates closely related to *Microcystis* spp. were the most abundant, with 52 unique candidate sequences representing 46% of total host reads.

**FIGURE 3:**
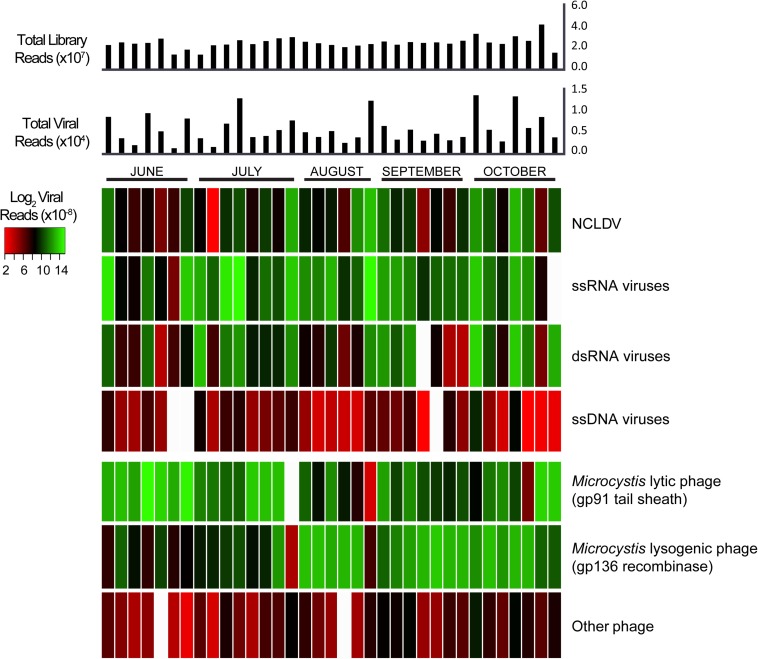
Heatmap of normalized viral abundances for each sample and virus type with histogram of total viral reads and total library reads in each sample. Each column describes a single sample. Color scale describes viral read abundance normalized to library size.

Our dataset also provided the opportunity to investigate variation in virus communities (based on transcript abundance for individual groups) over the course of the 5-month bloom. Figure 3 details the read abundances of each viral group we targeted, with each column representing a different sample and each row representing a different category of virus (alternative color scheme available, Supplementary Figure 6). The colors indicate the sum of viral transcripts for each viral type in each sample, normalized to individual library size. In some samples, the ssRNA viruses showed similar proportions to the *Microcystis* lytic and lysogenic phage infections, suggesting that these viruses might be just as active. Of these 35 samples, markers for *Microcystis* phage lytically infecting their host had the most abundant representation in 10 samples, *Microcystis* phage in a lysogenic infection had the most abundant representation in 15, ssDNA viruses had the most abundant representation in 9, and dsRNA viruses had the most abundant representation in one sample (Supplementary Figure 7). According to a Pearson correlation analysis, this pattern was best explained from the environmental data sets (Tang et al., 2018) by pH and salinity (Supplementary Figure 7A).

**FIGURE 4:**
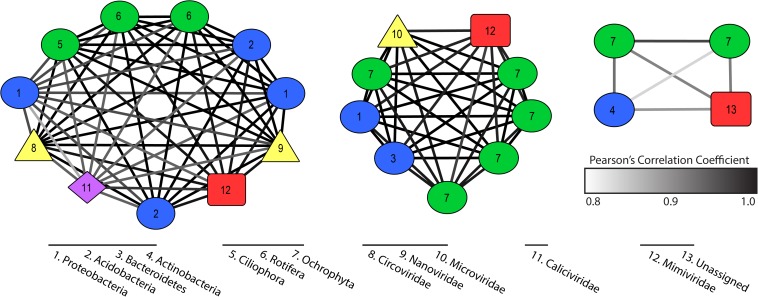
Co-occurrence clusters that contain both eukaryotic and bacterial hosts with one or more viruses. Colored symbols indicate host or virus type and edges describe strength of Pearson’s correlation. Numbers describe phylum identity of hosts and family identity of viruses.

Co-occurrence networks described putative virus and host interactions across our 35 samples. We established 20 significant co-occurrence clusters that ranged in membership from 2 to 11 members, with each network containing at least one putative virus and putative host. Twelve clusters contained a bacterial host with an RNA virus or a NCLDV, while four clusters contained a eukaryotic host with an RNA virus, a NCLDV, or ssDNA virus (Supplementary Table 4). Three clusters had both bacterial and eukaryotic hosts with a combination of virus types (Figure 4). An additional three clusters contained a virophage but no NCLDV, which are typically thought to co-infect eukaryotic host cells. In all three cases, the virophage co-occurred with an RNA virus (Supplementary Table 4).

The gene marker approach used in this study allowed us to characterize the viral community within a cyanobacterium-dominated HAB. This technique extended existing virus-finding programs, such as VirSorter or VirFinder, powerful tools which are currently limited to phage and metagenomic analysis (Roux et al., 2015; Ren et al., 2017). While a similar approach has been used to study marine eukaryotic phytoplankton systems and even *Sphagnum* peat bogs (Moniruzzaman et al., 2017; Stough et al., 2018), it has not been used to explore freshwater cyanobacterial blooms, to our knowledge. Our results are consistent with the *Aureococcus*-dominated HAB transcriptome analyzed by Moniruzzaman et al. (2017) in relation to the presence of both NCLDVs and RNA viruses. Both studies showed abundant *Mimiviridae* and extended *Mimiviridae* group members, although Moniruzzaman et al. (2017) did not distinguish between the two groups. Our results also showed a similar, smaller representation of *Asfarviridae* and *Phycodnaviridae*. Our ssRNA virus results are consistent with previous work that observed putative transcripts/viruses in this group, although several of *Marnaviridae* viruses were labeled “unclassified marine viruses” in the previous publication (Moniruzzaman et al., 2017). That article did not discuss dsRNA viruses, so it is unclear if our observed abundance (28% of RNA viral reads) of *Picobirnaviridae* and *Partitiviridae* is unusual. We also observed many virophage candidates, most of whom were closely related to the DSLV meta-assembly (a lake approximately 60 miles from our study site) (Gong et al., 2016).

Previously the viral component of metatranscriptomic analyses of *Microcystis* blooms have focused on the *Microcystis* phage (Steffen et al., 2017; Stough et al., 2017). However, our findings indicate that gene markers for RNA viruses, particularly ssRNA viruses, can reach similar transcript abundances during a bloom event. In many of our early season samples (June and July), we see higher relative abundances of ssRNA virus transcripts than the *Microcystis* lytic phage. These viruses are mostly members of *Marnaviridae*, whose members can infect photosynthetic eukaryotes including *C. tenuissimus*, *R. setigera*, *and Heterosigma akashiwo* (Nagasaki et al., 2004; Shirai et al., 2008). This suggests that these “neglected” viruses might play a role in the transition from a winter diatom bloom to the summer cyanobacterial blooms. Seasonal successions of this nature have been well-recorded in Lake Erie, another shallow lake plagued by summer *Microcystis* blooms (Saxton et al., 2012). Other virus-driven community controls have been hypothesized to occur in Lake Erie, with viral infection of parasitic fungal hosts hypothesized to contribute to the maintenance of winter diatom blooms (Edgar et al., 2016). In both scenarios, viral suppression of a predator or competitor enables a particular species or group to thrive.

A virus-mediated transition from diatoms to cyanobacteria can be explained within the context of a modified version of the “kill-the-winner” framework. As originally proposed by Thingstad and Lignell (Thingstad and Lignell, 1997), the “kill-the-winner” hypothesis posits that competition specialists – groups that would typically achieve high abundance due to abilities to assimilate resources and grow faster – are held at low densities when top-down pressure is strong (Thingstad and Lignell, 1997). This theory has been invoked to explain why heterotrophic bacteria do not outcompete phytoplankton when mineral nutrients, but not carbon, are limiting (Thingstad and Lignell, 1997). It has also been widely invoked as a mechanism to maintain diversity among bacteria (Winter et al., 2010; Kirchman, 2016), and it has been suggested that this framework is not limited to heterotrophic bacteria and might be applied to other co-occurring species in aquatic systems (Våge et al., 2013). Yet, it is not widely thought of in the context of phytoplankton bloom dynamics.

Silica may act as the constraining nutrient that catalyzes the transition of the community. Reductions in silica deposition are hypothesized to occur in association with increased pH conditions during *Microcystis* blooms, constraining diatom growth (Krausfeldt et al., 2019). In marine environments, silica limitation has been linked to increases in viral infection of diatoms (Kranzler et al., 2019). Acting together, nutrient limitation and viral infection could act as a negative feedback loop on diatoms that might provide an advantage for competitive species such as *Microcystis.*

The kill-the-winner hypothesis assumes equilibrium dynamics, and therefore is not implicated (or perhaps even appropriate) in selection during transient phytoplankton blooms. Bloom forming species are often thought of as opportunists, able to take advantage of ephemeral increases in resource availability, predominately through rapid cell division (Grover, 1990; Litchman and Klausmeier, 2001). In classical quantitative ecological theory, feedbacks on the nutrient environment and seasonal changes in environmental conditions control successional bloom dynamics, from opportunists to other groups (Margalef, 1978). In *Taihu*, diatoms are the opportunists. Abundant viruses consistent with those that infect dominant groups like *C. tenuissimus* and *R. setigera* imply that early diatom dominance may be terminated and subsequently suppressed by virus-mediated lysis. Collapse of diatoms would lead to opportunities for other groups, especially groups able to resist predation and infection. In this interpretation of the “kill-the-winner” hypothesis (Thingstad and Lignell, 1997), the potential for virus-induced cell lysis of the “neglected” community members could be one of the factors that indirectly allow for cyanobacterial bloom species to achieve dominance.

The latter season dominance of *Microcystis* depends on an ability to both consume the available nutrients and itself resist viral infection. One mechanism of *Microcystis* resistance is through high number of restriction modification systems (Zhao et al., 2018), but *Microcystis* may also minimize losses by forming a lysogenic state (Stough et al., 2017). In other bacterial systems, lysogeny can cause homoimmunity and allow microbes to escape secondary infection (*aka* super-infection) by other phage, ultimately prolonging the existence of the host (Donnelly-Wu et al., 1993). In the case of the *Microcystis* populations, this generates an ability for cells to escape (at least temporarily) the significant top down pressure from free *Microcystis*-infecting phage.

While we observed transcripts for viruses potentially capable of controlling a variety of protists, we saw a surprisingly limited richness and abundance in phage. The majority of phage transcripts were associated with *Microcystis* phage, which was not a surprise, given the dominance of the *Microcystis* host. However, *Microcystis* blooms are commonly accompanied by an abundant heterotrophic bacterial microbiome, particularly proteobacteria, who can play a large role in nitrogen cycling in this system (Wilhelm et al., 2011; Krausfeldt et al., 2017). We expected to observe a corresponding virus community, yet non-*Microcystis* phage accounted for less than 1% of total virus reads. This contradicts previous findings that phage are the most active viruses in aquatic microbial systems (Weinbauer, 2004) although it is consistent with transcriptomic observations in at least one other environment (Stough et al., 2019). This disparity may be the result of underestimation of phage sequences, our methodology, or a disproportionate expression of RNA virus transcripts. We note that a number of phage candidate sequences were disqualified as false positives during the BLASTx check against the non-redundant database. We identified these sequences as putative prokaryotes and removed them from further analysis and quantification. However, even if every disqualified candidate was considered incorrectly annotated and included in our quantifications, there were still ∼5× more transcripts associated with the non-phage “neglected” viruses than with the non-*Microcystis* phage. Other possibilities, including much larger burst sizes associated with RNA viruses or increased expression of the Rdrp gene marker are also possible explanations. However, the literature on this subject is patchy, with little information available focusing on insect viruses. To normalize for variation in expression of RNA virus genes during infection, an isolated infection transcriptome would be required for each algal virus we found.

Regardless, there is precedent to our findings; a similar gene marker approach also revealed higher richness and transcript abundance associated with NCLDVs and RNA viruses in a metatranscriptomic study of the microbiome of *Sphagnum* peat moss relative to the diversity of markers for phage gene expression (Stough et al., 2018). Another nucleic acid weighting approach also indicated a higher abundance of RNA viruses compared to DNA viruses in aquatic systems (Steward et al., 2013). It is unclear if our results show contradicting biology or capture previously overlooked and abundant virus types such as NCLDVs and RNA viruses.

Our co-occurrence analyses revealed populations that behave (in terms of persistence and abundance) in similar manners over the course of the bloom. We resolved 23 clusters that contained both host and virus transcripts with a statistically significant co-occurrence of expression across all 35 samples. While this is not directly indicative of a viral infection of the host in the cluster, it does imply these groups were responding to similar pressures in the system, either abiotic or biotic, and to some degree, their ecologies are thus “linked”. For example, we discovered several virophage that clustered significantly with RNA viruses and ssDNA viruses with no obvious NCLDV candidate. As virophage have only ever been identified co-infecting a host with a NCLDV, the observed clusters likely do not represent active co-infections, but hypothetically two different viruses benefiting from similar (or parallel) scenarios. We also observe clusters that contain only bacterial hosts and NCLDVs, the latter of which are to date only known to infect eukaryotes. Again, these co-occurrence observations are likely describing community dynamics (i.e. microbiomes) as opposed to direct infections. These smaller clusters of viruses and potential hosts can be used to generate hypotheses regarding predator-prey cascades in a community and interspecies viral interactions.

Given the above information, we looked for data and relationships consistent with the hypothesis that non-*Microcystis* viruses were involved in the establishment and/or maintenance of the *Microcystis* bloom. We observed two statically significant co-occurrences including cyanobacterial species and a *Marnaviridae-*like virus (consistent with viruses that infect *C. tenuissimus* and *R. setigera*) (Supplementary Table 4). By constraining competing members of the microbial community, these neglected viruses have the potential to create opportunities for *Microcystis* populations to compete for nutrient resources. Our findings highlight the activity of protist-infecting viruses – particularly ssRNA viruses – that have been overlooked. The evidence described here provides the rationale for future studies pertaining to the role of indirect infections influencing bloom-forming species, and the complicated collection of drivers that result in broad global persistence of *Microcystis*.

## Data Availability Statement

Raw sequencing data is publicly available in the MG-RAST database (Keegan et al., 2016) under the name “Lake_ *Taihu*_metatranscriptome_project.” Sequencing information and quality control details have been previously reported (Tang et al., 2018). Assembled data is also in the MG-RAST database under the name “*Taihu* 2014 MegaHit Assembly 1.”

## Author Contributions

HP and SW established the project direction. LK and XT performed the environmental sampling and subsequent RNA extractions. HP, EG, MH, and MS were responsible for computational processing. HP, DT, and SW were responsible for interpreting the data and composing the manuscript.

## Conflict of Interest

The authors declare that the research was conducted in the absence of any commercial or financial relationships that could be construed as a potential conflict of interest.

## References

[B1] Babicki S. Arndt D. Marcu A. Liang Y. Grant J. R. Maciejewski A. (2016). Heatmapper: web-enabled heat mapping for all. *Nucleic Acids Res.* 44 W147–W153. 10.1093/nar/gkw419 27190236 PMC4987948

[B2] Brussaard C. P. D. Kuipers B. Veldhuis M. J. W. (2005). A mesocosm study of *Phaeocystis globosa* population dynamics. *Harmful Algae* 4 859–874. 10.1016/j.hal.2004.12.015

[B3] Bullerjahn G. S. McKay R. M. Davis T. W. Backer D. B. Boyer G. L. D’Anglada L. V. (2016). Global solutions to regional problems: collecting global expertise to address the problem of harmful cyanobacterial blooms. A Lake Erie case study. *Harmful Algae* 54 223–238. 10.1016/j.hal.2016.01.003 28073479 PMC5230759

[B4] Chinchar V. Hyatt A. Miyazaki T. Williams T. (2009). “Family Iridoviridae: poor viral relations no longer,” in *Lesser known Large dsDNA Viruses*, ed. Van Etten J. L., (Berlin: Springer), 123–170. 10.1007/978-3-540-68618-7_4 19216437

[B5] Clarke K. R. Gorley R. N. (2015). *PRIMER V7: User Manual/Tutorial.* Plymouth: PRIMER-E.

[B6] Claverie J.-M. Abergel C. (2018). Mimiviridae: an expanding family of highly diverse large dsDNA viruses infecting a wide phylogenetic range of aquatic eukaryotes. *Viruses* 10:506. 10.3390/v10090506 30231528 PMC6163669

[B7] Donnelly-Wu M. K. Jacobs W. R. Jr. Hatfull G. F. (1993). Superinfection immunity of mycobacteriophage L5: applications for genetic transformation of mycobacteria. *Mol. Microbiol.* 7 407–417. 8459767 10.1111/j.1365-2958.1993.tb01132.x

[B8] Edgar R. Morris P. Rozmarynowycz M. D’souza N. Moniruzzaman M. Bourbonniere R. (2016). Adaptations to photoautotrophy associated with seasonal ice cover in a large lake revealed by metatranscriptome analysis of a winter diatom bloom. *J Great Lakes Res.* 42 1007–1015.

[B9] Fischer M. G. Suttle C. A. (2011). A virophage at the origin of large DNA transposons. *Science* 332 231–234. 10.1126/science.1199412 21385722

[B10] Gaia M. Benamar S. Boughalmi M. Pagnier I. Croce O. Colson P. (2014). Zamilon, a novel virophage with Mimiviridae host specificity. *PLoS One* 9:e94923. 10.1371/journal.pone.0094923 24747414 PMC3991649

[B11] Gann E. R. Pound H. L. Wilhelm S. W. (2019). *Python Script: Extracting Aligned Portion of Viral Hallmark Genes from Blastx Sequence.* Available: https://github.com/Wilhelmlab/general-scripts/blob/master/Pound2019_Extract_aligned.py (accessed February 26, 2020).

[B12] Gong C. Zhang W. Zhou X. Wang H. Sun G. Xiao J. (2016). Novel virophages discovered in a freshwater lake in China. *Front. Microbiol.* 7:5. 10.3389/fmicb.2016.00005 26834726 PMC4722103

[B13] Grover J. P. (1990). Resource competition in a variable environment: phytoplankton growing according to Monod’s model. *Am. Nat.* 136 771–789.

[B14] Hewson I. O’Neil J. M. Dennison W. C. (2001). Virus-like particles associated with *Lyngbya majuscula* (Cyanophyta; Oscillatoriacea) bloom decline in Moreton Bay, Australia. *Aquat. Microb. Ecol.* 25 207–213.

[B15] Ke Z. Xie P. Guo L. (2008). Controlling factors of spring–summer phytoplankton succession in Lake Taihu (Meiliang Bay, China). *Hydrobiologia* 607 41–49.

[B16] Keegan K. P. Glass E. M. Meyer F. (2016). MG-RAST, a metagenomics service for analysis of microbial community structure and function. *Methods Mol. Biol.* 1399 207–233. 10.1007/978-1-4939-3369-3_13 26791506

[B17] Kirchman D. L. (2016). Growth rates of microbes in the oceans. *Annu. Rev. Mar. Sci.* 8 285–309. 10.1146/annurev-marine-122414-033938 26195108

[B18] Kranzler C. F. Krause J. W. Brzezinski M. A. Edwards B. R. Biggs W. P. Maniscalco M. (2019). Silicon limitation facilitates virus infection and mortality of marine diatoms. *Nat. Microbiol.* 4 1790–1797. 10.1038/s41564-019-0502-x 31308524

[B19] Krausfeldt L. E. Farmer A. T. Castro Gonzalez H. Zepernick B. N. Campagna S. R. Wilhelm S. W. (2019). Urea is both a carbon and nitrogen source for *Microcystis aeruginosa*: tracking 13C incorporation at bloom pH conditions. *Front. Microbiol.* 10:1064. 10.3389/fmicb.2019.01064 31164875 PMC6536089

[B20] Krausfeldt L. E. Tang X. van de Kamp J. Gao G. Bodrossy L. Boyer G. L. (2017). Spatial and temporal variability in the nitrogen cyclers of hypereutrophic Lake Taihu. *FEMS Microbiol. Ecol.* 93:fix024. 10.1093/femsec/fix024 28334116

[B21] La Scola B. Desnues C. Pagnier I. Robert C. Barrassi L. Fournous G. (2008). The virophage as a unique parasite of the giant mimivirus. *Nature* 455:100. 10.1038/nature07218 18690211

[B22] Letunic I. Bork P. (2019). Interactive Tree Of Life (iTOL) v4: recent updates and new developments. *Nucleic Acids Res.* 47 W256–W259. 10.1093/nar/gkz239 30931475 PMC6602468

[B23] Li D. Liu C.-M. Luo R. Sadakane K. Lam T.-W. (2015). MEGAHIT: an ultra-fast single-node solution for large and complex metagenomics assembly via succinct de Bruijn graph. *Bioinformatics* 31 1674–1676. 10.1093/bioinformatics/btv033 25609793

[B24] Litchman E. Klausmeier C. A. (2001). Competition of phytoplankton under fluctuating light. *Am. Nat.* 157 170–187. 10.1086/318628 18707270

[B25] Margalef R. (1978). Life-forms of phytoplankton as survival alternatives in an unstable environment. *Oceanol. Acta* 1 493–509.

[B26] Matsen F. A. Kodner R. B. Armbrust E. V. (2010). pplacer: linear time maximum-likelihood and Bayesian phylogenetic placement of sequences onto a fixed reference tree. *BMC Bioinformatics* 11:538. 10.1186/1471-2105-11-538 21034504 PMC3098090

[B27] Moniruzzaman M. Wurch L. L. Alexander H. Dyhrman S. T. Gobler C. J. Wilhelm S. W. (2017). Virus-host relationships of marine single-celled eukaryotes resolved from metatranscriptomics. *Nat. Commun.* 8:16054. 10.1038/ncomms16054 28656958 PMC5493757

[B28] Nagasaki K. Tomaru Y. Nakanishi K. Hata N. Katanozaka N. Yamaguchi M. (2004). Dynamics of *Heterocapsa circularisquama* (Dinophyceae) and its viruses in Ago Bay, Japan. *Aquat. Microb. Ecol.* 34 219–226. 17504475

[B29] Niu Y. Shen H. Chen J. Xie P. Yang X. Tao M. (2011). Phytoplankton community succession shaping bacterioplankton community composition in Lake Taihu, China. *Water Res.* 45 4169–4182. 10.1016/j.watres.2011.05.022 21684570

[B30] Pound H. Wilhelm S. (2019). Metatranscriptomic screening for genes of interest. *Protocols.io.* 10.17504/protocols.io.7vyhn7w

[B31] Qin B. Q. Zhu G. W. Gao G. Zhang Y. L. Li W. Paerl H. W. (2010). A drinking water crisis in Lake Taihu, China: linkage to climatic variability and lake management. *Environ. Manag.* 45 105–112. 10.1007/s00267-009-9393-6 19915899

[B32] Ren J. Ahlgren N. A. Lu Y. Y. Fuhrman J. A. Sun F. (2017). VirFinder: a novel k-mer based tool for identifying viral sequences from assembled metagenomic data. *Microbiome* 5:69. 10.1186/s40168-017-0283-5 28683828 PMC5501583

[B33] Roux S. Enault F. Hurwitz B. L. Sullivan M. B. (2015). VirSorter: mining viral signal from microbial genomic data. *PeerJ* 3:e985. 10.7717/peerj.985 26038737 PMC4451026

[B34] Saxton M. A. D’souza N. A. Bourbonniere R. A. McKay R. M. L. Wilhelm S. W. (2012). Seasonal Si: C ratios in Lake Erie diatoms—evidence of an active winter diatom community. *J. Great Lakes Res.* 38 206–211.

[B35] Shirai Y. Tomaru Y. Takao Y. Suzuki H. Nagumo T. Nagasaki K. (2008). Isolation and characterization of a single-stranded RNA virus infecting the marine planktonic diatom *Chaetoceros tenuissimus* Meunier. *Appl. Environ. Microbiol.* 74 4022–4027. 10.1128/AEM.00509-08 18469125 PMC2446501

[B36] Smoot M. E. Ono K. Ruscheinski J. Wang P. L. Ideker T. (2011). Cytoscape 2.8: new features for data integration and network visualization. *Bioinformatics* 27 431–432. 10.1093/bioinformatics/btq675 21149340 PMC3031041

[B37] Steffen M. M. Belisle B. S. Watson S. B. Boyer G. L. Wilhelm S. W. (2014). Review: Status, causes and consequences of cyanobacterial blooms in Lake Erie. *J. Great Lakes Res.* 40 215–225.

[B38] Steffen M. M. Davis T. W. McKay R. M. L. Bullerjahn G. S. Krausfeldt L. E. Stough J. M. (2017). Ecophysiological examination of the Lake Erie *Microcystis* bloom in 2014: linkages between biology and the water supply shutdown of Toledo, OH. *Environ. Sci. Technol.* 51 6745–6755. 10.1021/acs.est.7b00856 28535339

[B39] Steward G. F. Culley A. I. Mueller J. A. Wood-Charlson E. M. Belcaid M. Poisson G. (2013). Are we missing half of the viruses in the ocean? *ISME J.* 7:672. 10.1038/ismej.2012.121 23151645 PMC3578568

[B40] Stough J. M. Tang X. Krausfeldt L. E. Steffen M. M. Gao G. Boyer G. L. (2017). Molecular prediction of lytic vs lysogenic states for *Microcystis* phage: metatranscriptomic evidence of lysogeny during large bloom events. *PLoS One* 12:e0184146. 10.1371/journal.pone.0184146 28873456 PMC5584929

[B41] Stough J. M. Yutin N. Chaban Y. V. Moniruzzaman M. Gann E. R. Pound H. L. (2019). Genome and environmental activity of a *Chrysochromulina parva* virus and its virophages. *Front. Microbiol.* 10:703. 10.3389/fmicb.2019.00703 31024489 PMC6459981

[B42] Stough J. M. A. Kolton M. Kostka J. E. Weston D. J. Pelletier D. A. Wilhelm S. W. (2018). Diversity of active viral infections within the *Sphagnum* microbiome. *Appl Environ. Microbiol.* 84:e1124-18. 10.1128/AEM.01124-18 30217851 PMC6238052

[B43] Tang X. Krausfeldt L. E. Shao K. LeCleir G. R. Stough J. M. Gao G. (2018). Seasonal gene expression and the ecophysiological implications of toxic *Microcystis aeruginosa* blooms in Lake Taihu. *Environ. Sci. Technol.* 52 11049–11059. 10.1021/acs.est.8b01066 30168717

[B44] Thingstad T. Lignell R. (1997). Theoretical models for the control of bacterial growth rate, abundance, diversity and carbon demand. *Aquat. Microb. Ecol.* 13 19–27.

[B45] Thingstad T. F. (2000). Elements of a theory for the mechanisms controlling abundance, diversity, and biogeochemical role of lytic bacterial viruses in aquatic systems. *Limnol. Oceanogr.* 45 1320–1328.

[B46] Tomaru Y. Tarutani K. Yamaguchi M. Nagasaki K. (2004). Quantitative and qualitative impacts of viral infection on a *Heterosigma akashiwo* (Raphidophyceae) bloom in Hiroshima Bay, Japan. *Aquat. Microb. Ecol.* 34 227–238.

[B47] Våge S. Storesund J. E. Thingstad T. F. (2013). Adding a cost of resistance description extends the ability of virus–host model to explain observed patterns in structure and function of pelagic microbial communities. *Environ. Microbiol.* 15 1842–1852. 23331773 10.1111/1462-2920.12077

[B48] Valles S. Chen Y. Firth A. Guérin D. A. Hashimoto Y. Herrero S. (2017). ICTV virus taxonomy profile: Dicistroviridae. *J. Gen. Virol.* 98 355–356. 10.1099/jgv.0.000756 28366189 PMC5797946

[B49] Vanderploeg H. A. Liebig J. R. Carmichael W. W. Agy M. A. Johengen T. H. Fahnenstiel G. L. (2001). Zebra mussel (*Dreissena polymorpha*) selective filtration promoted toxic *Microcystis* blooms in Saginaw Bay (Lake Huron) and Lake Erie. *Can. J Fish. Aquat. Sci.* 58 1208–1221.

[B50] Weinbauer M. G. (2004). Ecology of prokaryotic viruses. *FEMS Microbiol. Rev.* 28 127–181. 15109783 10.1016/j.femsre.2003.08.001

[B51] Wigington C. H. Sonderegger D. Brussaard C. P. D. Buchan A. Finke J. F. Fuhrman J. A. (2016). Re-examination of the relationship between marine virus and microbial cell abundances. *Nat. Microbiol.* 1:15024. 10.1038/nmicrobiol.2015.24 27572161

[B52] Wilhelm S. W. Farnsley S. E. LeCleir G. R. Layton A. C. Satchwell M. F. DeBruyn J. M. (2011). The relationships between nutrients, cyanobacterial toxins and the microbial community in Taihu (Lake Tai), China. *Harmful Algae* 10 207–215. 10.1016/j.hal.2010.10.001

[B53] Wilson W. H. Tarran G. A. Schroeder D. Cox M. Oke J. Malin G. (2002). Isolation of viruses responsible for the demise of an *Emiliania huxleyi* bloom in the English Channel. *J. Mar Biol. Assoc. U. K.* 82 369–377.

[B54] Winter C. Bouvier T. Weinbauer M. G. Thingstad T. F. (2010). Trade-offs between competition and defense specialists among unicellular planktonic organisms: the “killing the winner” hypothesis revisited. *Microbiol. Mol. Biol. Rev.* 74 42–57. 10.1128/MMBR.00034-09 20197498 PMC2832346

[B55] Zhao L. Song Y. Li L. Gan N. Brand J. J. Song L. (2018). The highly heterogeneous methylated genomes and diverse restriction-modification systems of bloom-forming *Microcystis*. *Harmful Algae* 75 87–93. 10.1016/j.hal.2018.04.005 29778228

[B56] Zhou J. Sun D. Childers A. McDermott T. R. Wang Y. Liles M. R. (2015). Three novel virophage genomes discovered from Yellowstone Lake metagenomes. *J. Virol.* 89 1278–1285. 10.1128/JVI.03039-14 25392206 PMC4300641

[B57] Zhou J. Zhang W. Yan S. Xiao J. Zhang Y. Li B. (2013). Diversity of virophages in metagenomic data sets. *J. Virol.* 87 4225–4236. 10.1128/JVI.03398-12 23408616 PMC3624350

