# Supplementary material for: The “Neglected Viruses” of Taihu: Abundant Transcripts for Viruses Infecting Eukaryotes and Their Potential Role in Phytoplankton Succession
Source: Front Microbiol. 2020 Mar 6;11:338. doi: 10.3389/fmicb.2020.00338 (PMC7067694; doi:10.3389/fmicb.2020.00338)
Supplement: Supplementary file 1 [file Data_Sheet_1.PDF]

Supplemental figures to be published online in *Frontiers in Microbiology* with

THE "NEGLECTED VIRUSES" OF *TAIHU*: ABUNDANT TRANSCRIPTS FOR VIRUSES INFECTING EUKARYOTES AND THEIR POTENTIAL ROLE IN PHYTOPLANKTON SUCCESSION

Helena L. Pound[1], Eric R. Gann[1], Xiangming Tang[2], Lauren E. Krausfeldt[1], Matthew Huff[3], Margaret E. Staton[3], David Talmy[1] and Steven W. Wilhelm[1]*

Department of Microbiology, The University of Tennessee, Knoxville TN 37996 USA
State Key Laboratory of Lake Science and Environment, Nanjing Institute of Geography & Limnology, Chinese Academy of Sciences, Nanjing, PR China
Department of Entomology and Plant Pathology, The University of Tennessee, Knoxville TN 37996 USA

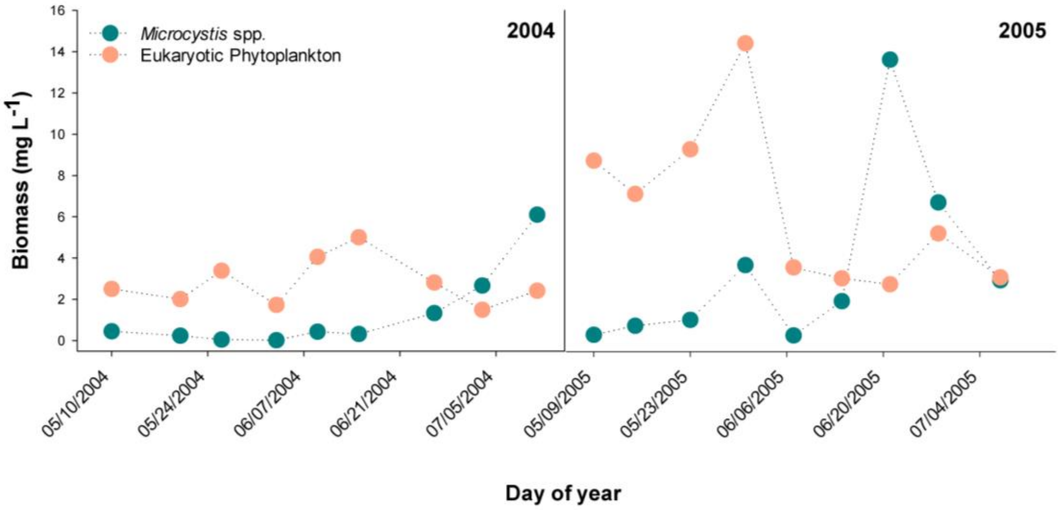

Supplemental Figure 1. Changes in biomass of *Microcystis* spp. And eukaryotic phytoplankton in Lake Taihu in 2004 and 2005. Adapted from Ke *et al.* 2008.

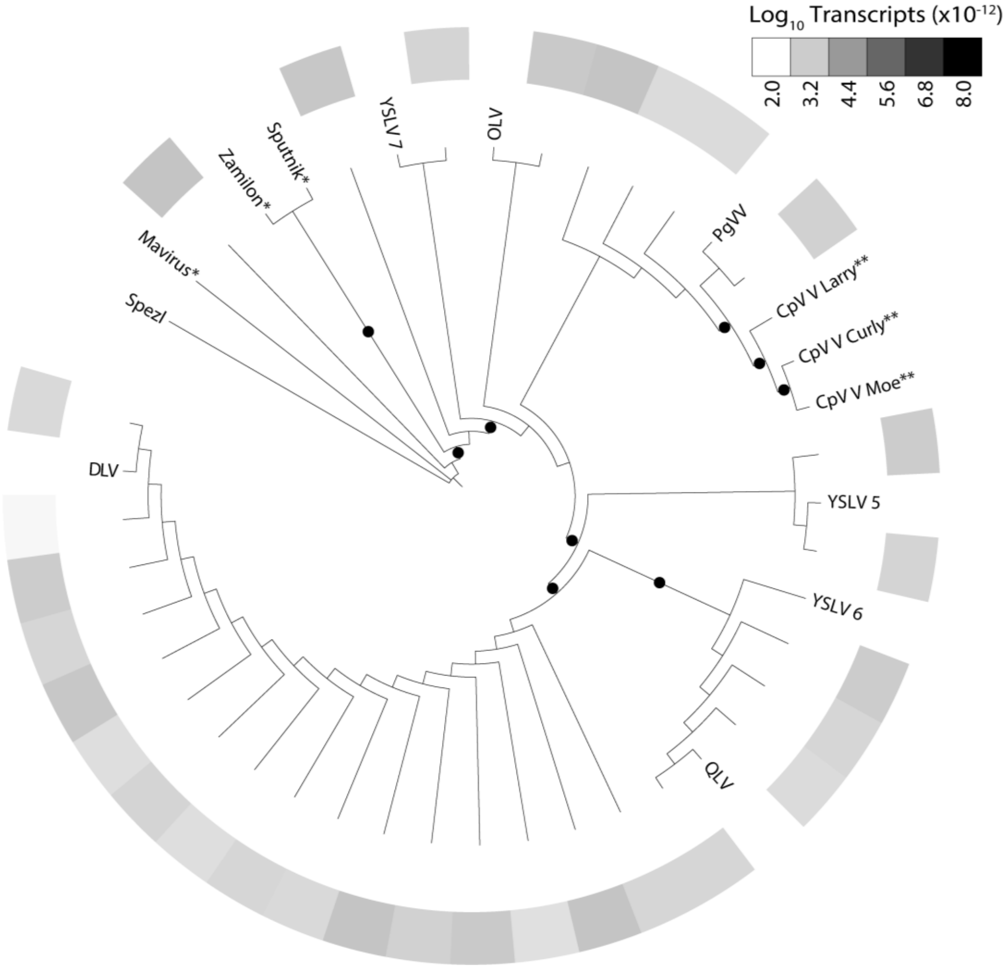

Supplemental Figure 2.
Maximum-likelihood phylogenetic placement of virophage candidates. Reference proteins are denoted by abbreviated names. Outer heatmap ring indicates normalized transcript abundance for each candidate summed for all samples. Black dots represent bootstrap values >0.5. *Isolated reference with genome sequenced. **Isolated with only environmental sequencing

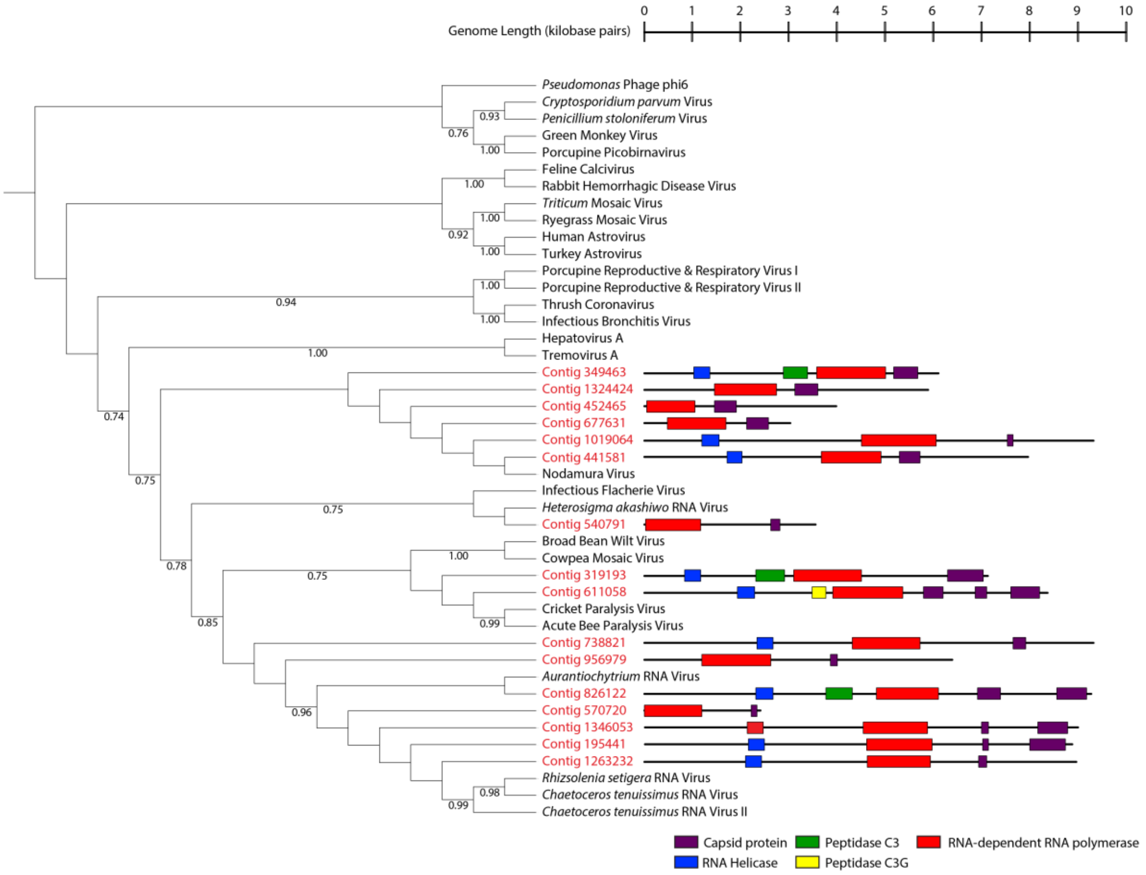

Supplemental Figure 3.
Maximum-likelihood phylogenetic placement of near full-length RNA virus genomes with genomic content (B). Reference proteins are denoted by abbreviated names in black. Candidates with near full-length genomes are denoted in red (B).

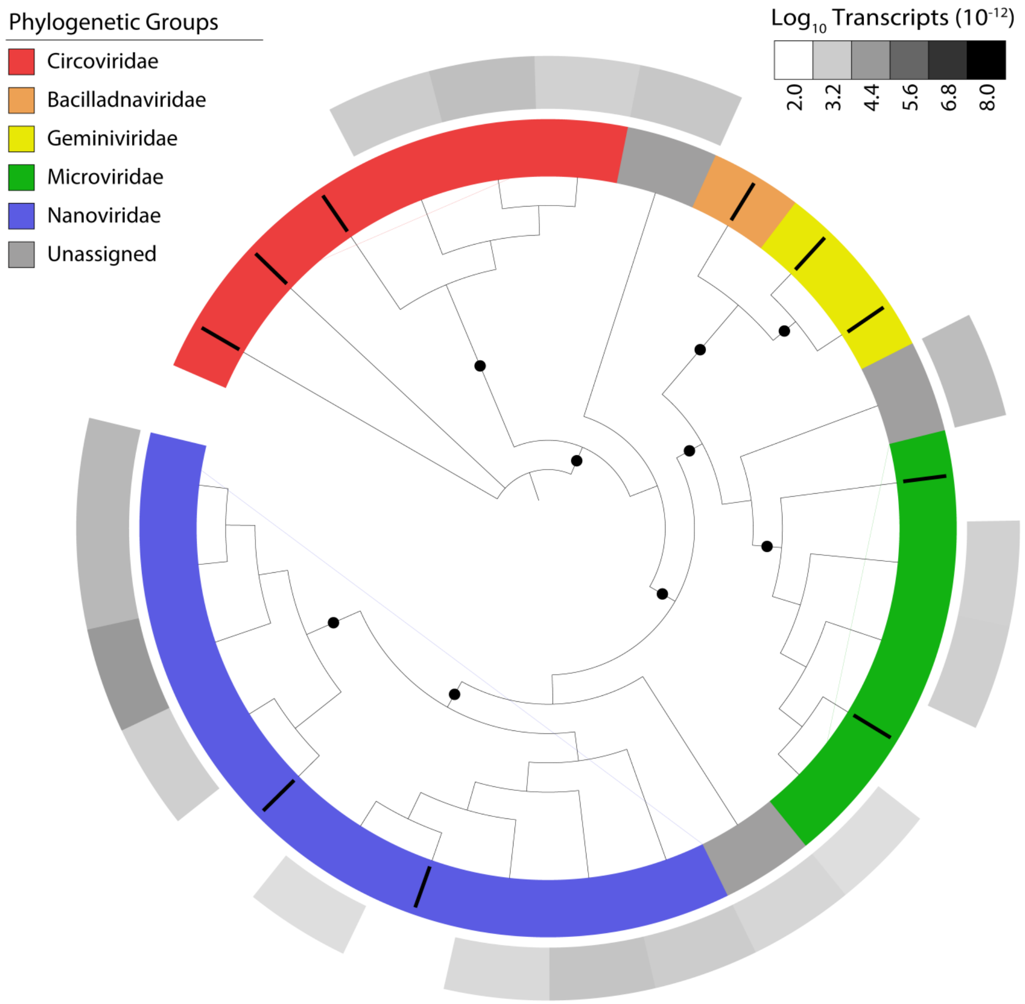

Supplemental Figure 4.
Maximum-likelihood phylogenetic placement of ssDNA virus candidates. Reference proteins are denoted by black bars with colors indicating viral family. The outer heatmap ring indicates normalized transcript abundance for each candidate summed for all samples. Black dots represent bootstrap values >0.5.

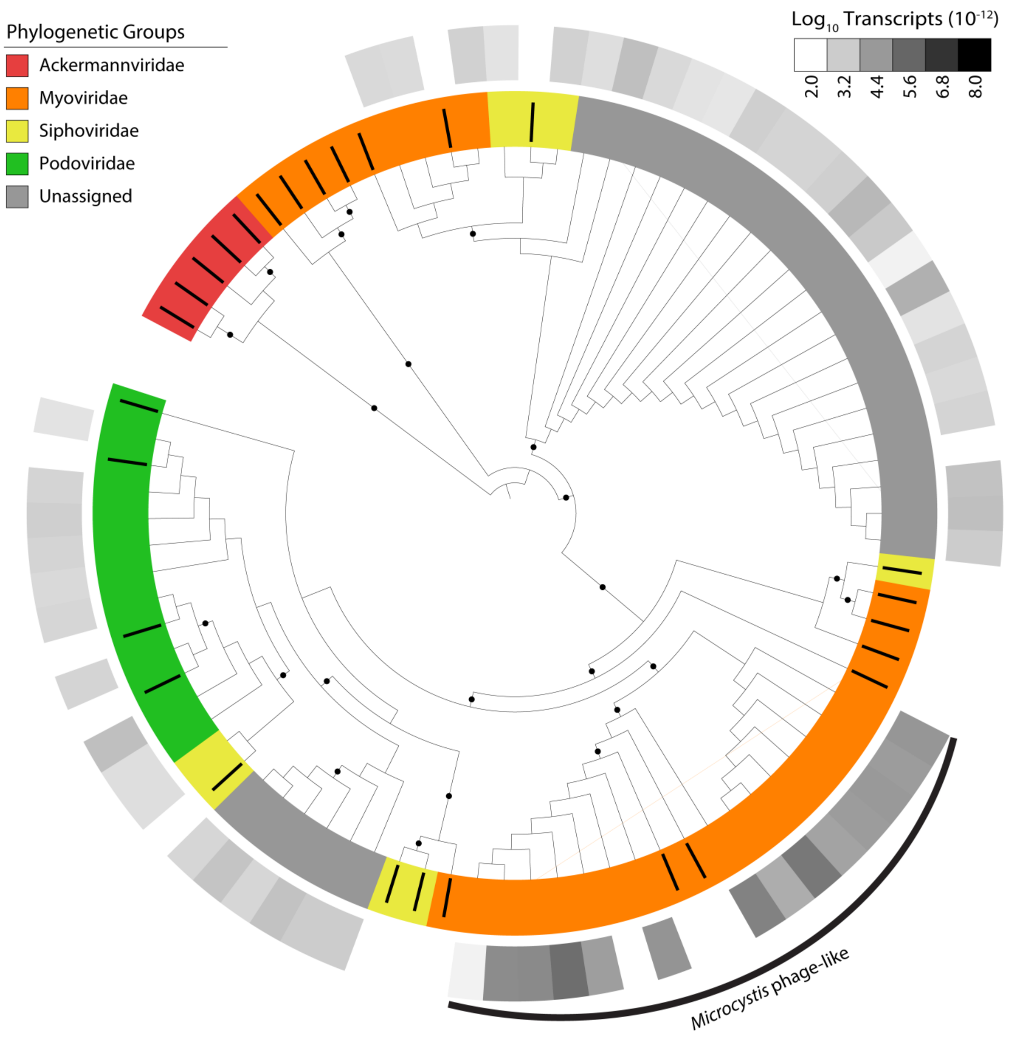

Supplemental Figure 5.
Maximum-likelihood phylogenetic placement of phage candidates. Reference proteins are denoted by black bars with colors indicating viral family. The outer heatmap ring indicates normalized transcript abundance for each candidate summed for all samples. Black dots represent bootstrap values >0.5.

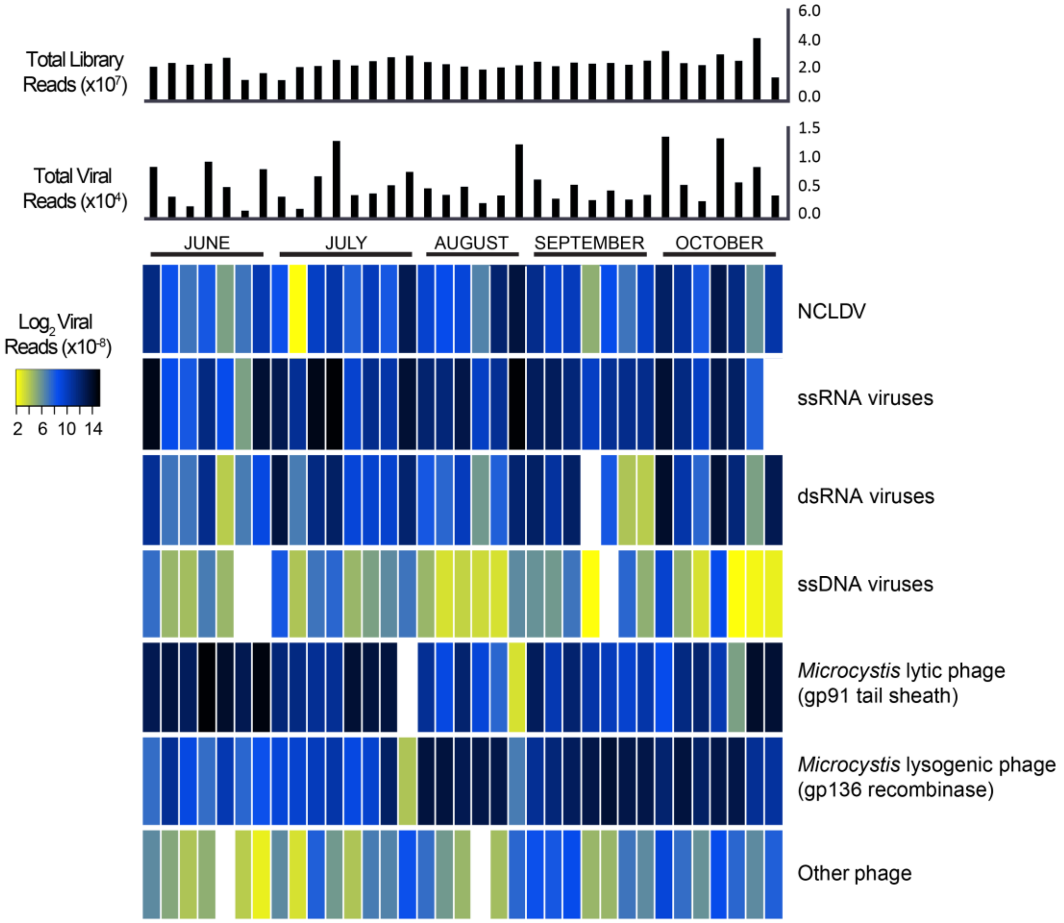

Supplemental Figure 6.
Alternative color scheme for heatmap of normalized viral abundances for each sample and virus type with histogram of total viral reads and total library reads in each sample. Each column describes a single sample. Color scale describes viral read abundance normalized to library size.

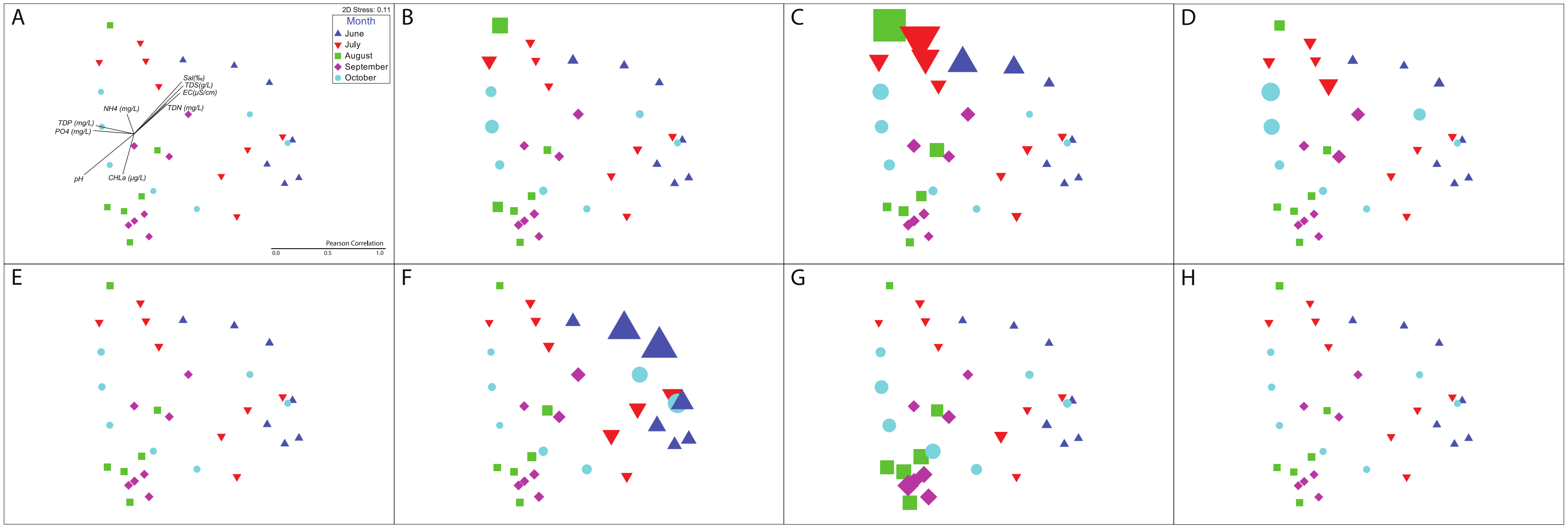

Supplemental Figure 7.
Non-metric multidimensional scaling plot describing Bray-Curtis dissimilarity between 35 samples. Colors indicate month of sampling. Vectors indicate environmental variables that explain nMDS positioning (A). B-H show the same nMDS with each sample scaled to show the abundance of a particular virus type in each sample: NCLDV (B), ssRNA virus (C), dsRNA virus (D), ssDNA virus (E), *Microcystis* lytic phage (F), *Microcystis* lysogenic phage (G), “other” phage (H).
